# Hemodynamic Collapse and Hidden Pathology: A Case of Perforated Duodenal Diverticulum Identified During Trauma Laparotomy

**DOI:** 10.7759/cureus.98518

**Published:** 2025-12-05

**Authors:** Bryan A Wallace, Jillian Lane, Victoria M Estevez, Daniel Shlyak, Angela Jones

**Affiliations:** 1 General Surgery, Delray Medical Center, Delray Beach, USA; 2 Medicine, St. George's University School of Medicine, True Blue, GRD; 3 Trauma Surgery, St. George's University School of Medicine, Delray Beach, USA; 4 Trauma Surgery, LECOM (Lake Erie College of Osteopathic Medicine) Bradenton, Delray Beach, USA; 5 Anesthesiology, Larkin Community Hospital, South Miami, USA; 6 General Surgery, Larkin Community Hospital, South Miami, USA; 7 Trauma Surgery, Delray Medical Center, Delray Beach, USA; 8 Critical Care, Delray Medical Center, Delray Beach, USA

**Keywords:** abdominal blunt trauma, duodenal diverticulum, emergency exploratory laparotomy, perforation, trauma

## Abstract

Duodenal diverticula are rare extraluminal sac-like protrusions of the duodenal wall. Perforation of the diverticula is a life-threatening but uncommon complication, particularly following blunt abdominal trauma. We present the case of an 82-year-old woman who sustained blunt abdominal trauma after being struck by a motor vehicle, resulting in perforation of a duodenal diverticulum. She was hemodynamically unstable on arrival, necessitating emergent exploratory laparotomy during which a stapled diverticulectomy was performed. This case highlights the rarity of traumatic perforation of duodenal diverticula and the challenges of diagnosing and managing such injuries in the acute setting. It also underscores the importance of flexible and timely surgical intervention, particularly when preoperative imaging is unavailable.

## Introduction

Duodenal diverticula are extraluminal sac-like protrusions of the duodenal wall [1]. They are broadly categorized as either congenital (true diverticula), which contain all layers of the intestinal wall - mucosa, submucosa, muscularis propria, and serosa - or acquired (false or pseudodiverticula), which consist only of mucosa and submucosa and lack the muscularis propria layer [2]. False diverticula occur most frequently in the colon, with the duodenum being the second most common site [3]. The exact cause of an acquired duodenal diverticulum (DD) remains unclear. Still, it is thought to result from abnormalities in peristalsis (synchronized contractions used to propel contents through the gastrointestinal tract) and elevated segmental intraluminal pressures [2]. Duodenal diverticula are present in approximately 5-10% of the population, most often arising on the medial wall of the second portion of the duodenum, near the ampulla of Vater [4]. While 95% of cases are asymptomatic, these diverticula can occasionally cause complications such as hemorrhage, biliary obstruction, inflammation, and most critically, perforation [4,5]. Duodenal perforation is a life-threatening event with high morbidity and mortality [6]. Approximately 162 cases of perforated duodenal diverticula have been reported in the literature [5]. Most reported cases of rupture occur spontaneously or result from iatrogenic injury during procedures such as endoscopic retrograde cholangiopancreatography (ERCP) [5], whereas traumatic perforation following blunt abdominal trauma is exceedingly rare. This case underscores the difficulty of accurate diagnosis and the need for decisive operative decision-making in the management of intra-abdominal injuries in unstable trauma patients, and it contributes to the limited literature on duodenal diverticular perforation secondary to blunt force trauma.

## Case presentation

An 82-year-old female pedestrian was struck by a motor vehicle and brought to the emergency department by emergency medical services transport as a trauma alert. On arrival, the primary survey was notable for tachypnea (35 bpm), hypotension (blood pressure 74/49), tachycardia (115 bpm), and a diminished level of consciousness (Glasgow Coma Scale 11/15). The secondary survey was notable for multiple deformities of her left lower extremity. The airway was secured with a cuffed endotracheal tube. The focused assessment with sonography (FAST) exam was negative. The portable X-ray was negative for cardiopulmonary pathology but positive for fractures of the bilateral pubic rami, left femur, tibia, and fibula. Laboratory workup revealed a hemoglobin of 11.6 g/dL, white blood count of 13.4 x 10^3^/mcL, platelet count of 256 x 10^3^/mcL, creatinine of 0.8 mg/dL, partial thromboplastin time (PTT) of 23.4 seconds, international normalized ratio (INR) of 1.1, and a prothrombin time (PT) of 11.9 seconds (Table 1). The patient was resuscitated with Ringer’s lactate solution but remained hypotensive. 

**Table 1:** Vitals and laboratory values upon arrival *Institution-specific reference range.

	Parameter	Value	Reference Range*	Units
Vitals				
	Heart Rate	115	60-100	Beats Per Minute
	Systolic Blood Pressure	74	90-120	Millimeters of Mercury (mmHg)
	Diastolic Blood Pressure	49	60-90	Millimeters of Mercury (mmHg)
	Respiratory Rate	35	12-18	Breaths Per Minute
	Oxygen Saturation	100	95-100	%
Labs				
	White Blood Cell	13.4	5-10	x10^3^/mcL
	Hemoglobin	11.6	14-18	g/dL
	Hematocrit	35.7	42-52	%
	Platelets	256	150-450	x10^3^/mcL
	Internal Normalized Ratio (INR)	1.1	0.8-1.2	None
	Prothrombin Time (PT)	11.9	9-11.5	Seconds (s)
	Partial Thromboplastin Time (PTT)	23.4	22.1-31.1	Seconds (s)
	Creatinine	0.8	0.6-1.3	mg/dL

The abdomen was opened in a standard fashion from the xiphoid process to the pubic symphysis. Upon entry into the peritoneum, no free fluid or active bleeding was identified. The abdomen was systematically explored, and pockets of air were found trapped under the gastrocolic ligament. The lesser sac was entered, revealing a retroperitoneal hematoma. A perforated hollow structure was identified, presumed to be the duodenum. The 2 cm defect was digitized, and bile was noted on the glove. This structure tapered down to a small opening. Further dissection revealed that this structure was a lateral outpouching of the second/third portion of the duodenum. The perforation was splayed open in a rosebud fashion with easily distinguishable mucosa.

**Figure 1:**
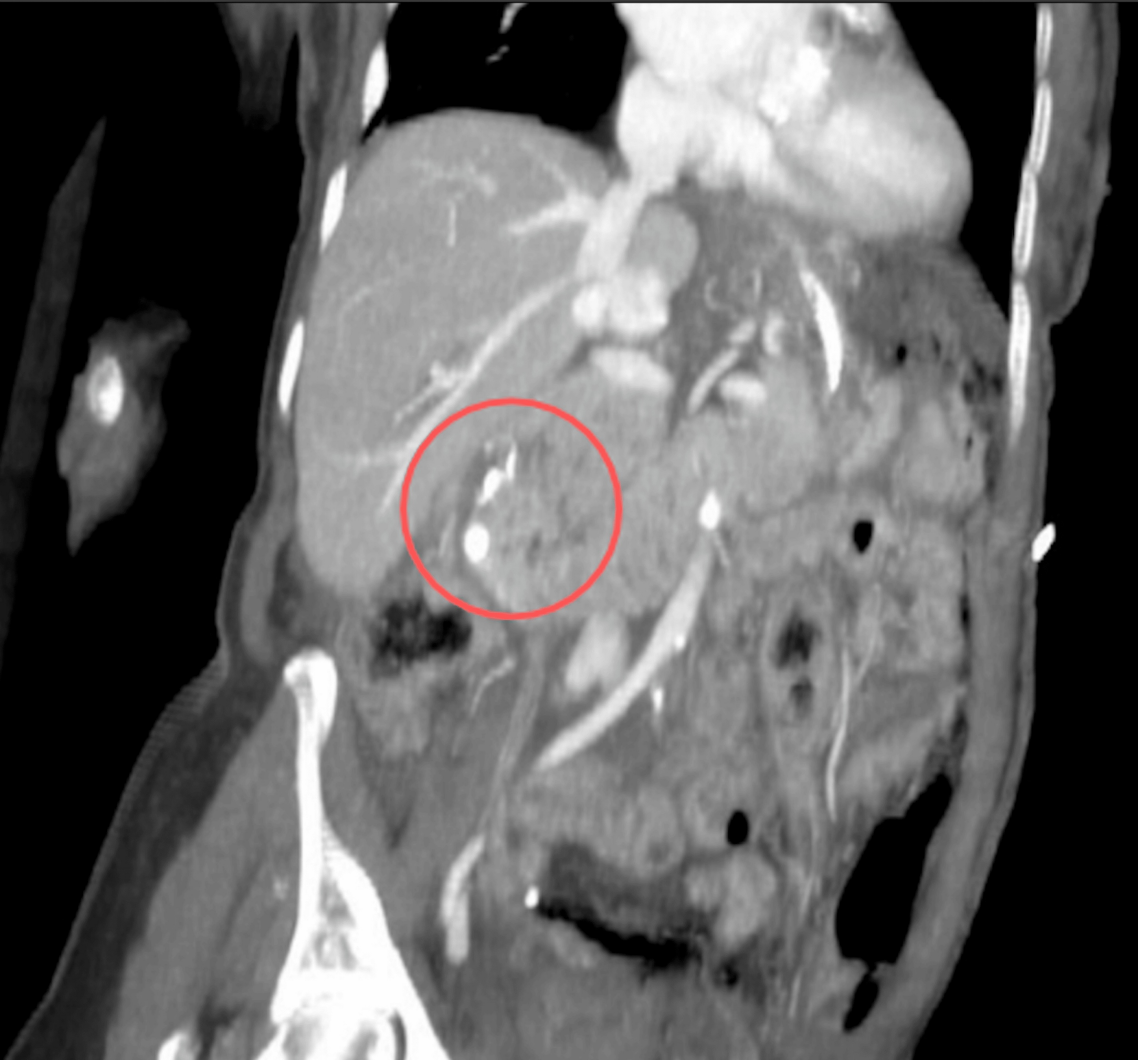
Oblique-angled computed tomography angiogram (CTA) of the abdomen and pelvis demonstrating the second and third portions of the duodenum, with the lateral staple line highlighted by the red circle.

Given the location and findings, the duodenum was mobilized via the Kocher maneuver, and the head of the pancreas was inspected, revealing a small hematoma without overt injury. This was followed by an intraoperative cholangiogram to rule out common bile duct injury. The cholangiogram was negative for leak but did not show flow of contrast into the duodenum, likely due to the inflammatory process. Diverticulectomy was then performed using a linear cutter stapler, leaving 1 cm of remaining diverticulum. Manual compression of the duodenum adjacent to the stapled diverticulum confirmed patency. No other injuries were identified. The abdomen was then temporarily closed, and the patient was transferred to the intensive care unit (ICU) for further resuscitation. Postoperative computed tomography imaging confirmed the diagnosis of DD (Figure 1).

On hospital day 2, the patient remained hypotensive despite aggressive resuscitation, required vasopressors, and frank blood was evacuated from the abdomen. Second-look laparotomy revealed bleeding from the falciform ligament and ischemia of the mid-jejunum. A 14 cm segment of small bowel was resected, the patient was left in discontinuity (leaving the jejunum unconnected), and the abdomen was temporarily closed. Third-look laparotomy was performed on hospital day 4, during which the small bowel was anastomosed and the abdomen was closed. The patient was unable to be weaned from the ventilator and was discharged to a long-term acute care hospital (LTAC) on hospital day 21. The patient was lost to follow-up despite multiple attempts to contact the patient at the LTAC facility.

## Discussion

We report a case of perforated DD in a trauma patient who was hemodynamically unstable on arrival. Initial diagnosis was complicated by the location of the DD in the second/third portion of the duodenum, as bleeding or free air is often trapped in the retroperitoneum. Bedside imaging, including X-ray and FAST exam, does not adequately assess the retroperitoneum, necessitating a low threshold for operative exploration if injury to retroperitoneal structures is suspected. Traumatic injuries to the abdomen can be masked by shock (low blood flow to organs causing ischemic injury), exacerbation of chronic disease, or distracting injuries. Per the Advanced Trauma Life Support (ATLS) guidelines in the setting of blunt abdominal trauma with refractory hypotension and no clear source of hemorrhage, further imaging was bypassed and the patient was taken emergently for exploratory laparotomy for presumed hemorrhagic shock secondary to intra-abdominal bleeding [7]. 

In stable, non-traumatic patients, perforated DD often presents with vague symptoms such as fever, right upper quadrant pain, nausea, and vomiting; in some instances, signs of peritonitis may occur [5,6]. Given the retroperitoneal location of much of the duodenum, initial complaints can also include back pain [6]. Both physical examination and laboratory tests may yield inconclusive results, complicating timely diagnosis. When there is high clinical suspicion, an oral and IV contrast-enhanced computed tomography (CT) scan of the abdomen and pelvis, particularly in axial or coronal views, is an essential diagnostic modality used to identify diverticular perforation [8]. In approximately two-thirds of cases, duodenal diverticula perforate into the retroperitoneal space from the second part of the duodenum. CT findings may demonstrate extraintestinal air in the retroperitoneum, para-duodenal region, and/or a leak of gastrointestinal contrast [9]. Unfortunately, for hemodynamically unstable trauma patients, there is often insufficient time to perform a CT scan for diagnostic clarification.

Management of perforated DD remains non-standardized. With a reported mortality rate of 30%, a low index of suspicion should prompt surgical evaluation and, if warranted, intervention [6]. The approach to surgical management depends primarily on the patient’s clinical condition and the location of the diverticulum [10]. A variety of surgical procedures have been documented for managing DD, including diverticulectomy with closure of the duodenal defect (stapled or hand-sewn, single or multiple layers), the use of an omental patch, segmental duodenectomy with duodenojejunostomy, duodenal occlusion with biliary diversion, and pylorus-preserving Whipple procedure [10]. Choosing among these options relies heavily on intraoperative assessment, where the surgeon must determine whether primary repair is feasible or if segmental resection with reconstruction is necessary, based on tissue viability, contamination, and the diverticulum’s proximity to critical structures.

In our case, the most appropriate and time-efficient approach was a damage control laparotomy followed by diverticulectomy. The clearly defined patient anatomy allowed for primary repair using a linear cutter stapler, enabling rapid resection of the diverticulum. Similarly, Moysidis et al. reported a case of spontaneous perforated DD in which diverticulectomy using a linear stapler resulted in patient recovery and discharge after 10 days [10]. Although not a trauma case, their report suggests that diverticulectomy can be a time-efficient approach with favorable postoperative results. Other case reports, such as Yeh et al., describe diverticulectomy with a linear stapler as the primary management for spontaneous perforated DD in hemodynamically stable, non-traumatic patients. In their case, the abdomen was explored laparoscopically rather than via laparotomy [11]. While the surgical technique was similar, their patient underwent preoperative CT imaging and a minimally invasive approach.

In hemodynamically unstable trauma patients, damage control laparotomy is often essential for rapid diagnosis, source control, and hemorrhage management. Metcalf et al. reported a similar case of traumatic perforated DD and emphasized that patients with intractable symptoms, or those in emergent settings with high suspicion for perforated diverticula, should undergo prompt surgical intervention [12]. In their report, definitive repair with immediate abdominal closure was achieved after diverticulectomy, demonstrating that primary closure can be viable when intraoperative conditions are favorable. By contrast, our case required staged re-explorations due to ongoing hemodynamic instability and subsequent ischemic bowel, underscoring the need for flexibility in surgical planning when immediate closure is not feasible.

There is no standardized algorithm or classification system guiding the management of perforated DD. Most literature focuses on spontaneous or iatrogenic causes, with limited data addressing trauma-associated perforations [10,13]. In a recent systematic review, only a minority of patients underwent emergent surgery without prior imaging, reflecting the rarity of scenarios like ours, where clinical instability mandates immediate operative exploration [13]. The selection of surgical approach, laparoscopy versus laparotomy, must be individualized, taking into account the diverticulum’s location, the patient’s physiologic status, and radiologic findings if available. More extensive procedures, such as duodenal resection, bypass, or even pancreaticoduodenectomy, are reserved for cases where diverticulectomy is not feasible due to friability of adjacent tissue, proximity to the ampulla of Vater, or massive tissue destruction [14-16]. Our case reinforces the need for clinical vigilance and operative adaptability, especially when imaging is unavailable and intraoperative findings serve as the primary guide for treatment.

## Conclusions

Traumatic perforation of a DD is rare and has a high mortality rate, especially in unstable patients. In this case, immediate exploration of the abdominal cavity was necessitated by an unstable patient in the setting of polytrauma. A stapled diverticulectomy provided rapid control of the perforation while adhering to damage control principles, allowing for staged laparotomies to address evolving pathology. This case highlights the need for awareness of atypical anatomy, flexibility in surgical planning, and a standardized approach for duodenal diverticular perforations.
